# C3d-targeted complement inhibitors to correct complement dysregulation in aHUS patients

**DOI:** 10.3389/fimmu.2025.1620996

**Published:** 2025-06-20

**Authors:** Valeria Guaschino, Donata Santarsiero, Sara Gastoldi, Joshua M. Thurman, V. Michael Holers, Shelia M. Violette, Fei Liu, Kelly C. Fahnoe, Chiara Guarinoni, Ariela Benigni, Giuseppe Remuzzi, Marina Noris, Sistiana Aiello

**Affiliations:** ^1^ Istituto di Ricerche Farmacologiche Mario Negri IRCCS, Clinical Research Center for Rare Diseases Aldo e Cele Daccò and Centro Anna Maria Astori, Science and Technology Park Kilometro Rosso, Bergamo, Italy; ^2^ Department of Medicine, University of Colorado School of Medicine, Aurora, CO, United States; ^3^ Preclinical Research, Q32 Bio Inc., Waltham, MA, United States

**Keywords:** complement system, complement inhibitors, aHUS, endothelial cell (EC), thrombus formation

## Abstract

Atypical hemolytic uremic syndrome (aHUS) is a rare and severe thrombotic microangiopathy caused by genetic or acquired abnormalities leading to activation of the complement alternative pathway on cell surfaces. This process leads to endothelial dysfunction and microvascular thrombosis. The introduction of anti-C5 antibodies has dramatically improved aHUS prognosis; however, these treatments require regular intravenous infusions and block systemic complement activity, exposing the patient to risk of infections. Recently complement inhibitors have been developed to selectively bind injury-associated target molecules, thereby concentrating the drug at specific cellular or tissue sites while preserving systemic complement function. This study evaluated the local complement inhibitory activity of new molecules that exploit the natural localization of C3d at complement activation sites on cells: namely the anti-C3d monoclonal antibody 3d8b conjugated with the first 10 or 17 short consensus repeats (SCRs) of complement receptor 1 (CR1_1–10_ and CR1_1-17_, respectively) or the first 5 SCRs of complement factor H (FH_1-5_). To this purpose we tested their capability to block C3 deposition and C5b-9 formation on microvascular endothelial cells (HMEC-1) exposed to serum from patients with aHUS. We also assessed their ability to prevent loss of anti-thrombogenic properties in HMEC-1 pre-exposed to aHUS serum and then perfused with control blood. We demonstrate that anti-C3d-antibody conjugated with CR1_1-10_, or CR1_1-17_, or FH_1–5_ effectively prevented aHUS serum-induced complement activation on HMEC-1, outperforming their non-targeted soluble counterparts. The efficacy of C3 convertase inhibition varied depending on the complement inhibitory component (CR1_1-17_ > CR1_1-10_ > FH_1-5_). However, all the inhibitors successfully blocked C5 convertase activity and eliminated the pro-thrombogenic effects of aHUS patients’ serum. These findings support the potential of tissue-targeted complement inhibition as a novel, non-systemic therapeutic strategy for aHUS and other diseases characterized by localized complement dysregulation.

## Introduction

Atypical hemolytic uremic syndrome (aHUS) is a rare and severe form of thrombotic microangiopathy caused by genetic or acquired abnormalities of the alternative pathway (AP) of complement activation, leading to uncontrolled and sustained complement activation on cell surfaces (1, 2). This results in endothelial cell dysfunction and formation of microvascular thrombi, with a particular predilection for renal vasculature (1, 2). About 60% of patients with aHUS carry loss-of-function rare variants (RVs) in genes encoding complement regulatory proteins (complement factor H, CFH; complement factor I, CFI; MCP/CD46) or genomic rearrangements in CFH-CFHRs, or gain-of-function RVs in components of the AP C3 convertase (C3 and complement factor B, CFB), or anti-CFH autoantibodies (1).

The introduction of anti-C5 therapies (eculizumab or ravulizumab) has significantly improved aHUS management, allowing patients to maintain hematological remission and preserve renal function (3, 4). Despite these benefits, these anti-C5 treatments come with limitations. They are associated with high costs and require regular, prolonged hospital visits for drug administration. Moreover, by inhibiting the terminal complement products C5a and C5b-9, these therapies increase the risk of serious infections, particularly with encapsulated bacteria. Consequently, vaccination against *Neisseria meningitidis* is mandatory prior to the initiation of anti-C5 treatments, and in some cases, prophylactic antibiotic therapy is recommended throughout the duration of treatment (3).

The complement cascade is a key part of the innate immune system, acting as a primary line of defense against invading pathogens and playing a role in the clearance of damaged cells and tissue regeneration (5). Thus, a challenge for complement inhibitory therapeutics is to block complement deleterious effects without impairing its beneficial functions.

To address this, several complement inhibitors have been developed to selectively bind target molecules, thereby concentrating the drug at specific cellular or tissue sites (6). One recent approach involves bispecific antibodies, which inhibit complement activation on particular cells by bringing endogenous complement regulators (such as CFH or C4BP) close to specific cell surface antigens (7). A more focused strategy involves directing complement inhibitors precisely to sites of complement activation. All three complement pathways (classical, lectin or alternative) converge at the proteolytic cleavage of C3, by C3 convertases, resulting in the generation of C3a and C3b (5). C3b serves as a component of the C5 convertase, which subsequently cleaves C5 to produce the anaphylatoxin C5a and the membrane attack complex (C5b-9) (5). To regulate this process, C3b is inactivated by CFI through a cofactor-driven mechanism, being processed first into iC3b and then into C3dg and C3d. These fragments are deposited at high density on tissue surfaces via covalent binding at sites where complement activation has occurred.

By exploiting the natural localization of C3d at complement activation sites, various complement inhibitors have been developed through the conjugation of complement regulators to C3d-recognizing molecules, including the 4N-terminal short consensus repeats (SCRs) domains of complement receptor type 2 (8) and anti-C3d antibodies (9, 10). In this context, recent studies have described fusion proteins composed of a monoclonal antibody (mAb) raised against human C3d, linked to fragments of endogenous complement regulators containing their complement regulatory domains, specifically the first 10 SCRs of complement receptor type 1 (CR1) or the first 5 SCRs of CFH (9, 10). CR1 and CFH negatively regulate the complement AP by accelerating the decay of the C3 convertase and acting as cofactors for the proteolytic activity of CFI (5). The selected mAb clone, 3d8b, binds with high affinity to the C-terminal sequence of C3d, also present in the related fragments iC3b and C3dg (9). The term “anti-C3d” is used when describing the activity of the C3d mAb fusion proteins (9, 10). The mAb is linked to two moieties of either CR1_1–10_ or FH_1–5_ fragments, conferring a synergistic inhibitory effect. *In vivo* studies in experimental models of complement-mediated diseases have demonstrated that both anti-C3d-mAb-CR1_1–10_ and anti-C3d-mAb-FH_1–5_ constructs inhibit complement activity in tissues with localized complement activation at low concentrations that do not affect systemic complement activation (9, 10).

The objective of this study was to evaluate the effectiveness of the anti-C3d mAb 3d8b conjugated with the first 10 or 17 SCRs of CR1 (CR1_1–10_ and CR1_1–17_ including one and two C3b binding sites, respectively) or the first 5 SCRs of CFH (FH_1-5_), in inhibiting the uncontrolled activity of C3 and C5 convertases, as well as in preventing the loss of anti-thrombogenic properties induced ex-vivo on microvascular endothelial cells by serum from patients with aHUS, taken as prototype of cell-surface restricted complement-related diseases.

## Methods

### Study participants

Participants were 9 adult patients with aHUS in remission, included in the International Registry of Recurrent and Familial Haemolytic Uremic Syndrome/Thrombotic Thrombocytopenic Purpura (HUS/TTP). The Registry was established in 1996 at the Aldo e Cele Daccò Clinical Research Center for Rare Diseases (Ranica, Bergamo) (villacamozzi.marionegri.it/seu). Samples collected were stored at the Mario Negri Institute Biological Resources Center, in Biobank for Rare Diseases and Kidney Diseases (Ranica, Bergamo).

Three patients carry pathogenic RVs in *CFH* gene (#1, p.R1210C; #2, p.R78G; #3, p.S1191L), three patients in *C3* gene (#4, p.D61N; #5, p.S1063R; #6, p.T162R), and three patients in *CFI* gene (#7, p.P50R; #8, p.V412M; #9, p.C67W). No patients have anti-CFH autoantibodies. **Table 1** summarizes clinical and genetic characteristics of the patients. Rare functional variants (missense, nonsense, indel, or splicing variants) with minor allele frequency (MAF) <0.001 in the Genome Aggregation Database (gnomAD, https://gnomad.broadinstitute.org/) were selected. Stop-gain, frameshift and splicing variants, and missense variants with published functional studies, were categorized as pathogenic variants (PV). The other variants were categorized as likely pathogenic variants (LPV) or variants of uncertain significance (VUS), using guidelines from the American College of Medical Genetics and Genomics (ACMG) and from the KDIGO conference on aHUS and C3G (11–13).

**Table 1:** clinical and genetic characteristics of aHUS patients.

Patient	Platelets	LDH	Hemoglobin	C3	C4	Serum creatinin	Rare complement	gnomAD	Classification
	150-400.000/µl	266–500 IU/L	14–18 g/dL	70–152 mg/dL	15–40 mg/dL	0.55-1.25 mg/dL	Gene variants	Frequency	
1	302	488	14,6	105	35,4	3,81	CFH - p.R1210C	0.0003	PV
2	163	653	10,4	-	-	4,59	CFH - p.R78G	-	PV
3	328	453	11,4	63	33	8,11	CFH - p.S1191L	-	PV
4	302	303	11,5	47,7	27,5	8,9	C3 - p.D61N	0.00001	LPV
5	164	394	12,2	86	-	3,2	C3 - p.S1063R	8,11 x 10^-6^	PV
6	177	219	12,7	44	26	2,6	C3 - p.T162R	-	VUS
7	150	-	15	125	41	1,5	CFI - p.P50R	0.0001	VUS
8	209	430	11,6	-	-	6,7	CFI – p.V412M	0.0001	VUS
9	223	373	11,3	89	32,7	6,7	CFI - p.C67W	-	PV

PV, pathogenic variant; LPV, likely pathogenic variant; VUS, variant of uncertain significance.

aHUS was diagnosed in patients who have had one or more episodes of microangiopathic hemolytic anemia and thrombocytopenia, with hematocrit (Ht) <30%, hemoglobin (Hb) <10 g/dL, serum lactate dehydrogenase (LDH) >500 IU/L, undetectable haptoglobin, fragmented erythrocytes in the peripheral blood smear, and platelet count <150,000/μL, associated with acute renal failure (serum creatinine >1.3 mg/dL, and/or urinary protein/creatinine ratio >200 mg/g, or an increase in serum creatinine or a urinary protein/creatinine ratio >15% compared to baseline levels) (14). TTP was ruled out on the basis of ADAMTS13 activity >10% and no anti-ADAMTS13 antibodies. Stx-*E.Coli* infection was ruled out based on negative assays for *stx* and *eae* genes (by PCR) or Shiga-toxin (Vero cell assay) in the stool and/or anti-Shiga-toxin antibodies (ELISA) and/or LPS O157, O26, O111, or O145 (ELISA) in serum.

Full remission was defined as the normalization of both hematological parameters (Ht >30%; Hb >10 g/dL; LDH <500 IU/L; platelets >150,000/μL) and renal function (serum creatinine <1.3 mg/dL for adults, urinary protein/creatinine ratio <200 mg/g).

Patients had not taken C5 inhibitory drugs for at least 8 weeks prior to blood draws. The protocol was approved by the Ethical Committee of the Azienda Sanitaria Locale Bergamo, Italy.

### Evaluation of serum-induced C3 deposition on microvascular endothelial cells

The human microvascular endothelial cell line of dermal origin (HMEC-1, a kind gift of Dr. Edwin Ades and Francisco J. Candal of CDC and Dr. Thomas Lawley of Emory University, Atlanta, GA) was cultured as described (14, 15). For the test, HMEC-1 were plated on glass coverslips and used when confluent. Cells were washed three times with test medium (HBSS: 137 mmol/l NaCl, 5.4 mmol/l KCl, 0.7 mmol/l Na_2_HPO_4_, 0.73 mmol/l KH_2_PO_4_, 1.9 mmol/l CaCl_2_, 0.8 mmol/l MgSO_4_, 28 mmol/l Trizma base pH 7.3, 0.1% dextrose; with 0.5% BSA) and then activated with 10 µM ADP (Sigma-Aldrich) for 10 minutes. At the end, cells were washed and then incubated for 4 hours with aHUS serum or with a pool of 10 control sera (NHS) run in each experiment diluted 1:2 with test medium (50% of serum final concentration) in the presence or absence of: the pan-complement inhibitor sCR1 (150 µg/ml, i.e. 600 nM), anti-C3d mAb conjugated with CR1_1-10_ (1, 10, 100 nM, i.e. 0.290, 2.901, 29.008 µg/ml) or non-targeted CR1_1-10_ (1, 10, 100 nM, i.e. 0.073, 0.726, 7.258 µg/ml), anti-C3d mAb conjugated with CR1_1-17_ (1, 10, 100 nM, i.e. 0.389, 3.887, 38.874 µg/ml) or non-targeted CR1_1-17_ (1, 10, 100 nM, i.e. 0.122, 1.219, 12.191 µg/ml), anti-C3d mAb conjugated with FH_1-5_ (10, 100, 1000 nM, i.e. 2.171, 21.707, 217.068 µg/ml) or non-targeted FH_1-5_ (10, 100, 1000 nM, i.e. 0.361, 3.607, 36.072 µg/ml). **Table 2** summarizes structural characteristics of the complement inhibitors (anti-C3d mAb conjugated and unconjugated) used. The anti-C3d mAb, 3d8b, recognizes a shared epitope with iC3b, C3dg and C3d. The term “anti-C3d” is used when describing the activity of the C3d mAb fusion proteins.

**Table 2:** structural characteristics of anti-C3d mAb conjugated and unconjugated complement inhibitors.

Complement inhibitors	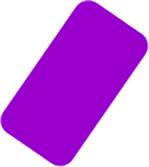	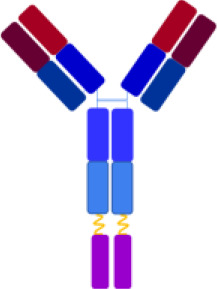	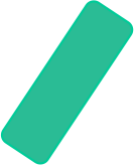	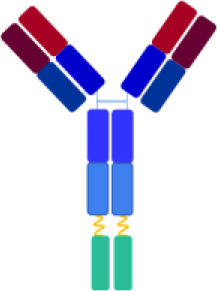	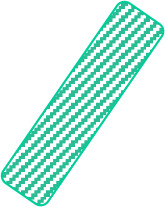	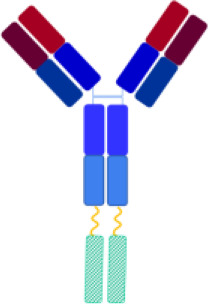
Targeting Domain	-	C3d-mAb	-	C3d-mAb	-	C3d-mAb
Linker	-	(G4S)_2_	-	(G4S)_2_	-	(G4S)_2_
Negative Regulator_SCRs_	fH_1-5_	fH_1-5_	CR1_1-10_	CR1_1-10_	CR1_1-17_	CR1_1-17_

At the end of the incubation step, HMEC-1 were washed, fixed in 3% paraformaldehyde, washed again and then blocked with PBS with 2% BSA for one hour. The cells were stained with FITC-conjugated rabbit anti-human C3c (Dako, that recognizes C3c, part of C3 and C3b, 1:300 final dilution in Dapi 1 µg/mL). In selected experiments, to confirm target presence on treated HMEC-1, cells have been stained with mouse anti-human C3d (3d8b, the same clone used for the anti-C3d conjugated complement inhibitors, 1.75 µg/ml) or the isotype control (mouse IgG1, k [MOPC-21] from murine myeloma, Sigma) followed by PE-conjugated rat anti-mouse light chain kappa secondary antibody (H139-52.1, Abcam, 1:100 final dilution in 1 µg/mL Dapi).

The fluorescent staining was acquired through the Apotome Axio Imager Z2 microscope (Zeiss) and the stained area was evaluated using Image J (NIH, Bethesda, MD). For each sample, 15 fields were analyzed, the highest and the lowest values were discarded and the mean was calculated on the remaining 13 fields.

Results were expressed in pixel^2^ or as percentage of stained area relative to area measured after exposure to aHUS serum alone, which was set as 100%. Half maximal inhibitory concentration (IC_50_) curves for C3d-targeted complement inhibitors were generated using non-linear regression in GraphPad PRISM v10 with normalized response (aHUS serum alone, 100%), values were expressed as mean ± SEM.

To assess whether the anti-C3d mAb (3d8b) alone, which targets complement inhibitors (CR1_1-10_, CR1_1-17_, and FH_1-5_) to C3d, affects complement activation and deposition on HMEC-1 cells incubated with aHUS serum, preliminary experiments were conducted using aHUS serum added with 3d8b mAb (10, 100, and 1000 nM). C3 deposits on ADP-activated HMEC-1 exposed to aHUS serum added with 3d8b mAb, were comparable to those observed with serum alone, indicating that the 3d8b mAb alone did not affect complement activation and C3 deposition on HMEC-1 exposed to aHUS serum (**Supplementary Figure 1**), and did not mask C3 detection on cells as well.

### Evaluation of serum-induced C5b-9 formation on microvascular endothelial cells

Experiments were performed as described above for C3 deposition evaluation. HMEC-1 cells were incubated with serum from aHUS patients in the presence or absence of anti-C3d mAb conjugated with CR1_1–10_ or non-targeted CR1_1-10_ (10 nM), anti-C3d mAb conjugated with CR1_1–17_ or non-targeted CR1_1-17_ (10 nM), anti-C3d mAb conjugated with FH_1–5_ or non-targeted FH_1-5_ (100, 1000 nM). At the end of the incubation step, fixed cells were stained with rabbit anti–human complement C5b-9 complex (Calbiochem, 1:200 final dilution) followed by FITC-conjugated secondary antibody (Jackson ImmunoResearch Laboratories, 1:50 final dilution in 1 µg/mL Dapi). The fluorescent staining was acquired through the Apotome Axio Imager Z2 microscope (Zeiss) and the stained area was evaluated using Image J (NIH, Bethesda, MD). For each sample, 15 fields were analyzed, the highest and the lowest values were discarded and the mean was calculated on the remaining 13 fields.

Results were expressed as stained area in pixel^2^.

### Evaluation of platelet adhesion and aggregation on HMEC-1 exposed to aHUS serum and then perfused with control blood

Platelet adhesion and aggregation on HMEC-1 under flow condition was performed as described previously (16). HMEC-1 were plated on a glass slide and used when they formed a monolayer. Cells were activated with ADP (10 μM) for 10 minutes and then exposed for 4 hours to serum (aHUS or control serum pool) diluted 1:2 with test medium, in the presence or absence of anti-C3d conjugated with CR1_1-17_ (10 nM) or with FH_1-5_ (100 and 1000 nM), or sCR1 (150 µg/ml), or eculizumab (100 µg/ml, based on recommended C_through_ target level of 50–100 µg/ml). After a quick flush with HBSS, HMEC-1 were perfused with heparinized (10 IU/ml heparin) whole blood from healthy subjects (pre-labeled with the fluorescent dye mepacrine 10 μM) in a thermostatic flow chamber (37°C). A constant flow rate of 1500 sec^-1^ (60 dynes/cm^2^) was applied to mimic the shear stress conditions found in the microcirculation (16). Three different healthy subjects were used as blood donors for all the experiments. The same blood donor was used for all the slides/conditions evaluated in each experiment. After 3 minutes, perfusion was stopped and the slide with the endothelial cell monolayer was dehydrated and fixed in acetone for 20 minutes. The integrity of the cell monolayer after the exposure to serum was verified in parallel slides in which cells were stained with May-Grunwald Giemsa.

The fluorescent staining was acquired through the Apotome Axio Imager Z2 microscope (Zeiss) and the stained area was evaluated using Image J (NIH, Bethesda, MD). For each sample, 15 fields were analyzed, the highest and the lowest values were discarded and the mean was calculated on the remaining 13 fields. Results were expressed as area covered by platelet aggregates, in pixel^2^.

### Statistical analysis

Data were reported as mean ± SD. The normality of value distribution was assessed using the D’Agostino-Pearson’s test. Comparisons of groups were performed with one-way analysis of variance followed by *post hoc* Student-Newman-Keuls test or Kruskal-Wallis test followed by *post hoc* Conover test, as appropriate. Statistically significant differences were assumed at a 5% level of probability. All calculations were made using MedCalc 10.0.1 statistical software (MedCalc Software, Mariakerke, Belgium).

## Results

### Deposition of C3 activation and degradation products on the surface of HMEC-1 exposed to serum from aHUS patients

We included in this study 9 aHUS patients in remission out of anti-C5 therapy who carried rare variants (RVs) in the *CFH*, or *CFI*, or *C3* genes. **Table 1** summarizes clinical and genetic characteristics of the patients.

**Figure 1:**
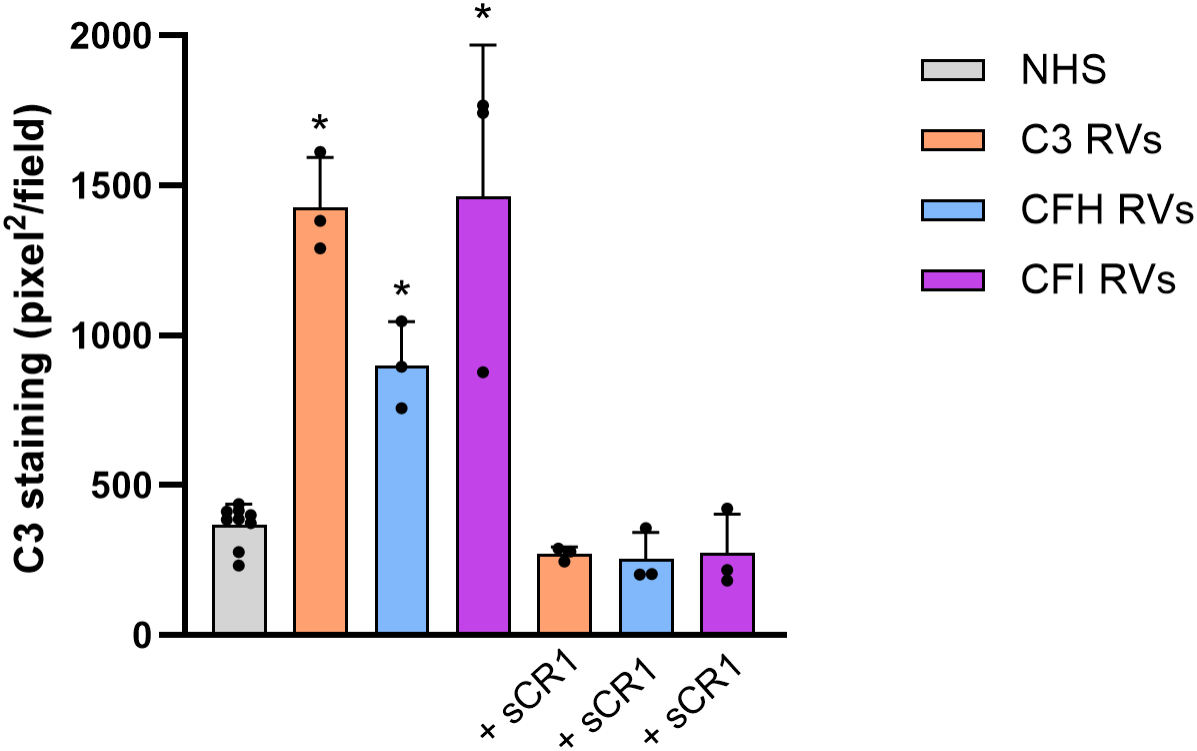
C3 deposition induced on activated HMEC-1 by serum from aHUS patients carrying RVs in *C3* (n=3), *CFH* (n=3) or *CFI* (n=3) genes. Data are expressed as area covered by C3 staining measured by anti-C3c mAb (mean ± SD). * p<0.05 vs NHS and corresponding +sCR1. RVs, rare variants; NHS, normal human serum.

Consistently with previously published reports (14–18), C3 deposition induced on ADP-activated HMEC-1 by serum from aHUS patients was significantly higher than that obtained with control serum pool, regardless of the genetic defect (**Figure 1**). C3 deposits were evaluated by anti-C3c antibody that recognizes an epitope shared by intact C3 and C3b. The addition of the pan-complement inhibitor sCR1 at the concentration of 150µg/ml (600 nM), which has been shown to completely block complement activation (16), fully prevented the effect of aHUS serum on C3 deposits (**Figure 1**). These data indicated that aHUS serum-induced C3 deposition entirely reflected complement activation.

To further confirm the above findings, we evaluated C3d deposited on ADP-activated HMEC-1 exposed to aHUS serum using the anti-C3d mAb 3d8b. This antibody recognizes a neoepitope shared by the complement fragments iC3b, C3dg, and C3d without binding to C3/C3b/C3c, and it has been documented to detect C3d deposition on biopsy specimens from various complement-related autoimmune kidney and skin conditions (9, 10). We observed a significant increase in C3d staining on ADP-activated cells exposed to aHUS serum compared to those exposed to control serum (**Supplementary Figure 2**).

### Anti-C3d mAb conjugated with CR1_1-10_, CR1_1–17_ or FH_1–5_ inhibit C3 deposition on HMEC-1 exposed to serum from aHUS patients

We then evaluated whether fusion proteins combining an anti-C3d monoclonal antibody (mAb) with complement inhibitors could efficiently block aHUS serum-induced complement activation on endothelial cell surface.

**Figure 2:**
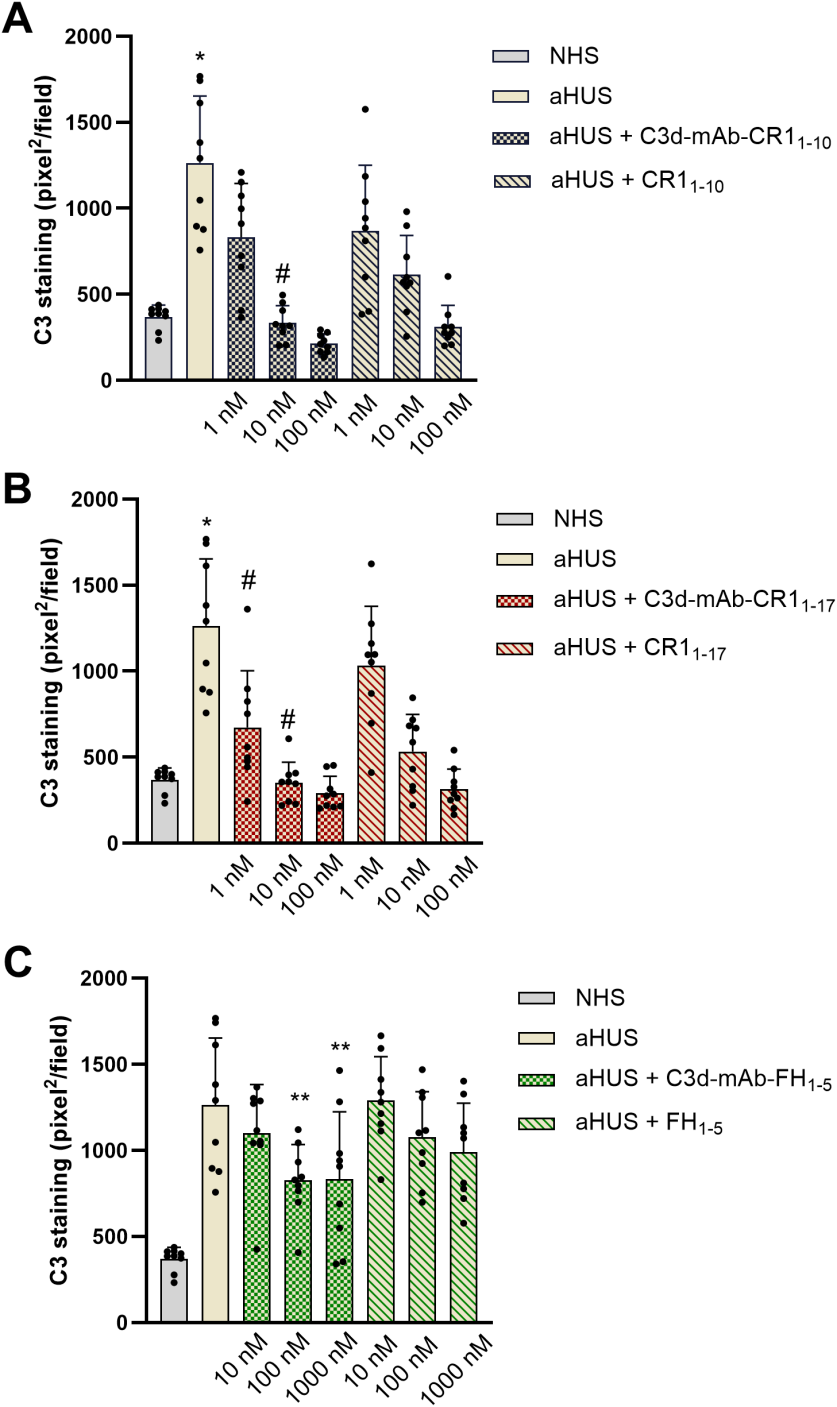
C3 deposition induced on activated HMEC-1 by serum from aHUS patients (three with *CFH* RVs, three with *CFI* RVs, and three with *C3* RVs) in the presence or absence of anti-C3d mAb conjugated with CR1_1-10_ (1, 10, 100 nM) or the corresponding unconjugated CR1_1-10_ (1, 10, 100 nM) **(A)**; anti-C3d mAb conjugated with CR1_1-17_ (1, 10, 100 nM) or the corresponding unconjugated CR1_1-17_ (1, 10, 100 nM) **(B)**; and anti-C3d mAb conjugated with FH_1-5_ (10, 100, 1000 nM) or the corresponding unconjugated FH_1-5_ (10, 100, 1000 nM) **(C)**. Data are expressed as area covered by C3 staining (mean ± SD, nine independent experiments). Each experiment includes cells exposed to NHS, or serum from a specific aHUS patient, with or without complement inhibitors. *p<0.05 vs all groups; #p<0.05 vs equimolar CR1_1–10_ or CR1_1-17_; **p<0.05 vs aHUS. NHS: normal human serum.

**Figure 3:**
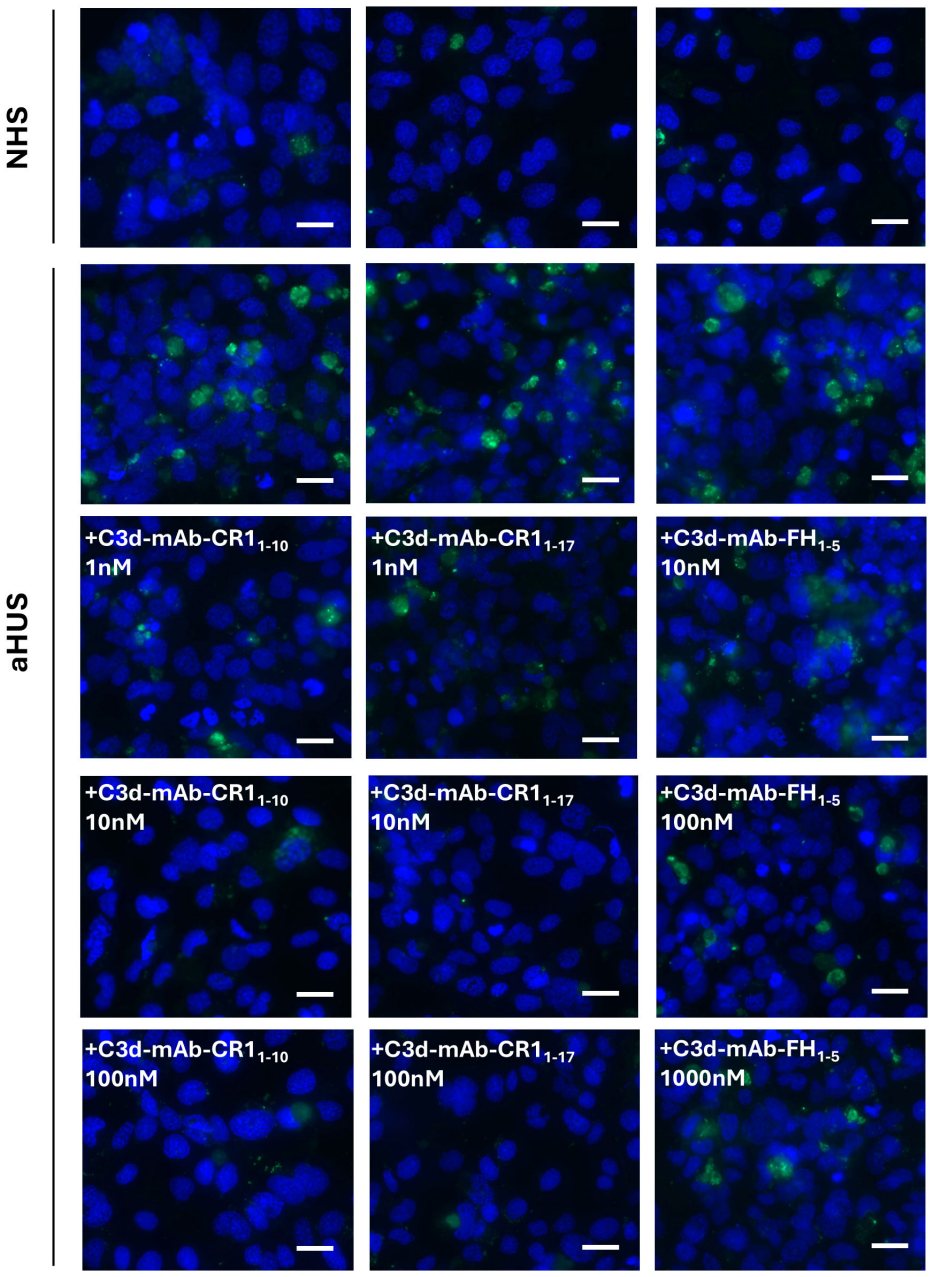
Representative images of C3 staining (green) on activated HMEC-1 exposed to normal human serum (NHS) or serum from aHUS patients in the presence or absence of anti-C3d-CR1_1-10_, anti-C3d-CR1_1-17_, and anti-C3d-FH_1-5_. Blue: DAPI. Scale bar: 20 µm.

Both anti-C3d mAb conjugated with CR1_1–10_ and soluble CR1_1–10_ added to serum from aHUS patients, dose dependently reduced C3 deposition on ADP-activated HMEC-1 (**Figures 2A**, **3**). Notably, at the dose of 10nM, C3d-targeted CR1_1–10_ was more effective than equimolar non-targeted CR1_1-10_ (**Figure 2A**). The anti-C3d mAb alone had no effect on aHUS serum-induced C3 deposition on HMEC-1, indicating that it did not independently influence complement activation on HMEC-1 exposed to aHUS serum and did not interact with anti-C3c Ab staining (**Supplementary Figure 1**).

Similar results were obtained with C3d-targeted CR1_1–17_ added to aHUS serum, although it was more effective in reducing C3 deposition than non-targeted CR1_1-17_ both at 1nM and 10 nM doses (**Figures 2B**, **3**).

Half maximal inhibitory concentrations (IC_50_) on C3 deposition were 2.61 ± 0.48 nM and 1.05 ± 0.60 nM for C3d-targeted CR1_1–10_ and C3d-targeted CR1_1-17_, respectively. Of note the IC_50_s of the non-targeted inhibitors were about 4-fold higher than the corresponding C3d-targeted ones (CR1_1-10_: 8.16 ± 1.96 nM; CR1_1-17_: 6.61 ± 1.67 nM).

At 10 nM concentration, neither the C3d-targeted FH_1-5_, which combines the anti-C3d mAb with the complement-regulatory domain of CFH (SCRs 1-5), nor the non-targeted FH_1–5_ reduced serum-induced C3 deposition on ADP-activated HMEC-1 (**Figures 2C**, **3**). Increasing the concentrations to 100 and 1000 nM, led to a partial but significant reduction of C3 deposits on HMEC-1 exposed to aHUS serum added with C3d-targeted FH_1-5_, while equimolar non-targeted FH_1–5_ had no effect (**Figures 2C**, **3**).

**Figure 4:**
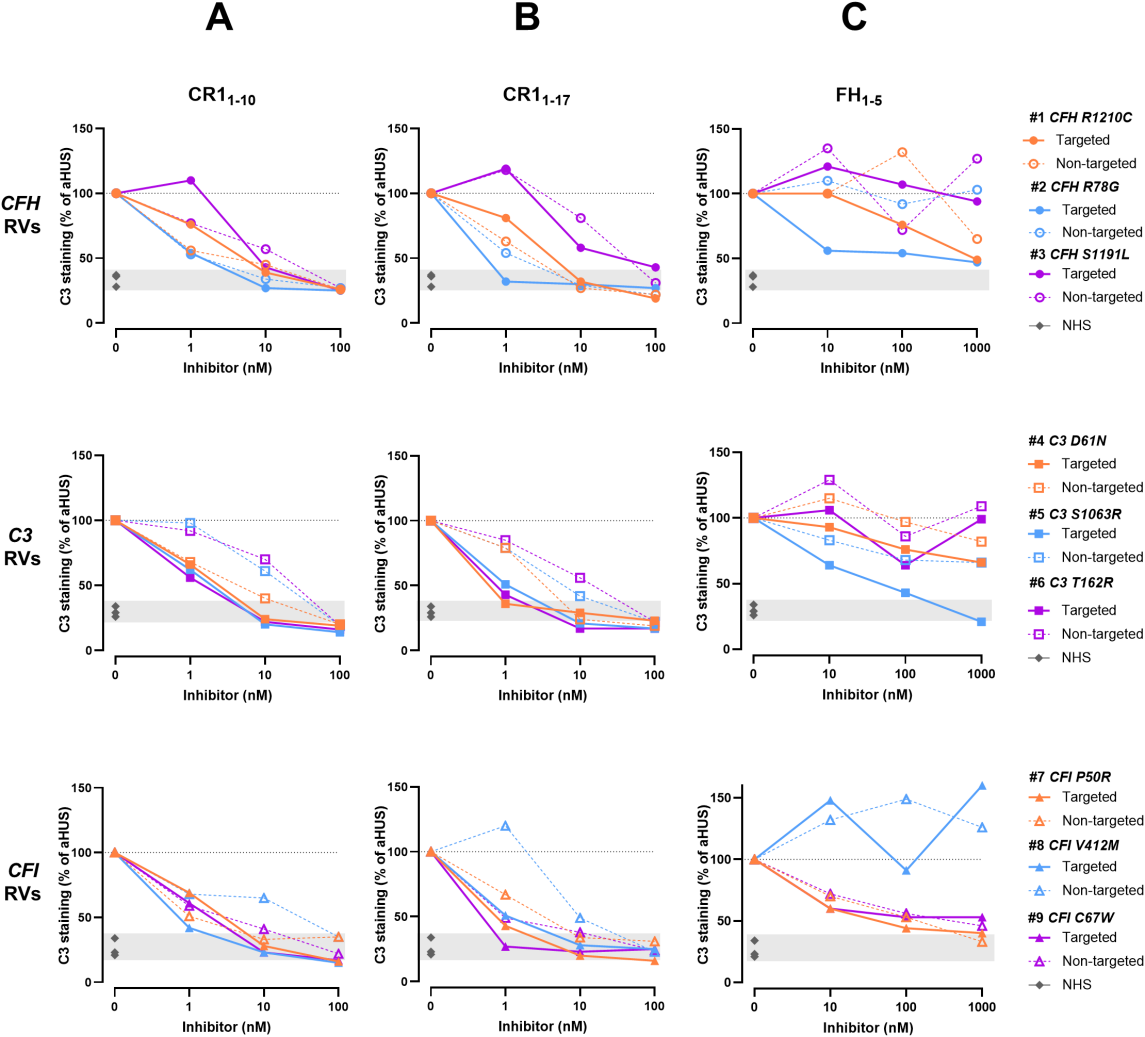
C3 deposition induced on activated HMEC-1 by serum from aHUS patients in the presence or absence of C3d-targeted or non-targeted CR1_1-10_ **(A)**, C3d-targeted or non-targeted CR1_1-17_ **(B)**, and C3d-targeted or non-targeted FH_1-5_ **(C)**. Data are expressed as percentages of serum-induced C3 deposition in the presence of complement inhibitors, relative to aHUS serum alone, which is set at 100%. Upper panels: *Patients carrying CFH RVs* (n=3 independent experiments). Each experiment includes cells exposed to NHS, or serum from a specific aHUS patient with or without complement inhibitors. Colored points represent the percentage value for individual patients/experiments. #1: patient carrying p.R1210C RV in *CFH* gene. #2: patient carrying p.R78G RV in *CFH* gene. #3: patient carrying p.S1191L RV in *CFH* gene. Middle panels: *Patients carrying C3 RVs* (n=3 independent experiments). Each experiment includes cells exposed to NHS, or serum from a specific aHUS patient with or without complement inhibitors. Colored points represent the percentage value for individual patients/experiments. #4: patient carrying p.D61N RV in *C3* gene. #5: patient carrying p.S1063R RV in *C3* gene. #6: patient carrying p.T162R RV in *C3* gene. Lower panels: *Patients carrying CFI RVs* (n=3 independent experiments). Each experiment includes cells exposed to NHS, or serum from a specific aHUS patient with or without complement inhibitors. Colored points represent the percentage value for individual patients/experiments. #7: patient carrying p.P50R RV in *CFI* gene. #8: patient carrying p.V412M RV in *CFI* gene. #9: patient carrying p.C67W RV in *CFI* gene. RVs, rare variants; NHS, normal human serum. Dashed line: 100% aHUS serum.

To compare the inhibitory potential of C3d-targeted complement inhibitors among patients with RVs in different genes, we expressed the results as percentage of C3 deposition induced by aHUS serum in the presence of complement inhibitors, relative to C3 deposition in their absence, which was set to 100%. The analysis of individual data revealed that, at the 1 nM dose, C3 deposition was minimally or not prevented on HMEC-1 exposed to sera from patients #1 and #3 – carrying the p.R1210C and p.S1191L RVs in *CFH* gene, respectively – with either C3d-targeted CR1_1–10_ or C3d-targeted CR1_1-17_ (**Figures 4A, B**). At the 10nM and 100nM doses, C3 deposits were nearly and completely in the normal range, respectively (**Figures 4A, B**). On the other hand, patients with either *C3* or *CFI* RVs were more sensitive to the inhibitory effects of both C3d-targeted CR1_1–10_ and C3d-targeted CR1_1-17_ (**Figures 4A, B**). As for fusion proteins combining anti-C3d mAb with FH_1-5_, the analysis of results in individual patients evidenced that a subgroup of patients was more sensitive to the inhibitory effect of C3d-targeted FH_1-5_. Indeed, the compound was able to substantially reduce ex-vivo serum-induced C3 deposition even at the dose of 10 nM in patient #2 carrying the p.R78G RV in *CFH* gene, in patient #5 carrying the p.S1063R RV in *C3* gene, and in patients #7 and #9, who carry the p.P50A and p.C67W RVs in *CFI* gene (**Figure 4C**).

### Anti-C3d mAb conjugated with CR1_1-10_, CR1_1–17_ or FH_1–5_ inhibit C5b-9 formation on HMEC-1 exposed to serum from aHUS patients

As in aHUS the terminal product of the complement cascade is strongly activated on endothelium (14, 15), we then tested the efficacy of C3d-targeted complement inhibitors in preventing C5b-9 formation induced by aHUS serum on HMEC-1. For 3 patients, namely patient #1 (p.R1210C RV in *CFH*), #5 (p.S1063R RV in *C3*) and #7 (p.P50A RV in *CFI*), there were sufficient serum samples available.

**Figure 5:**
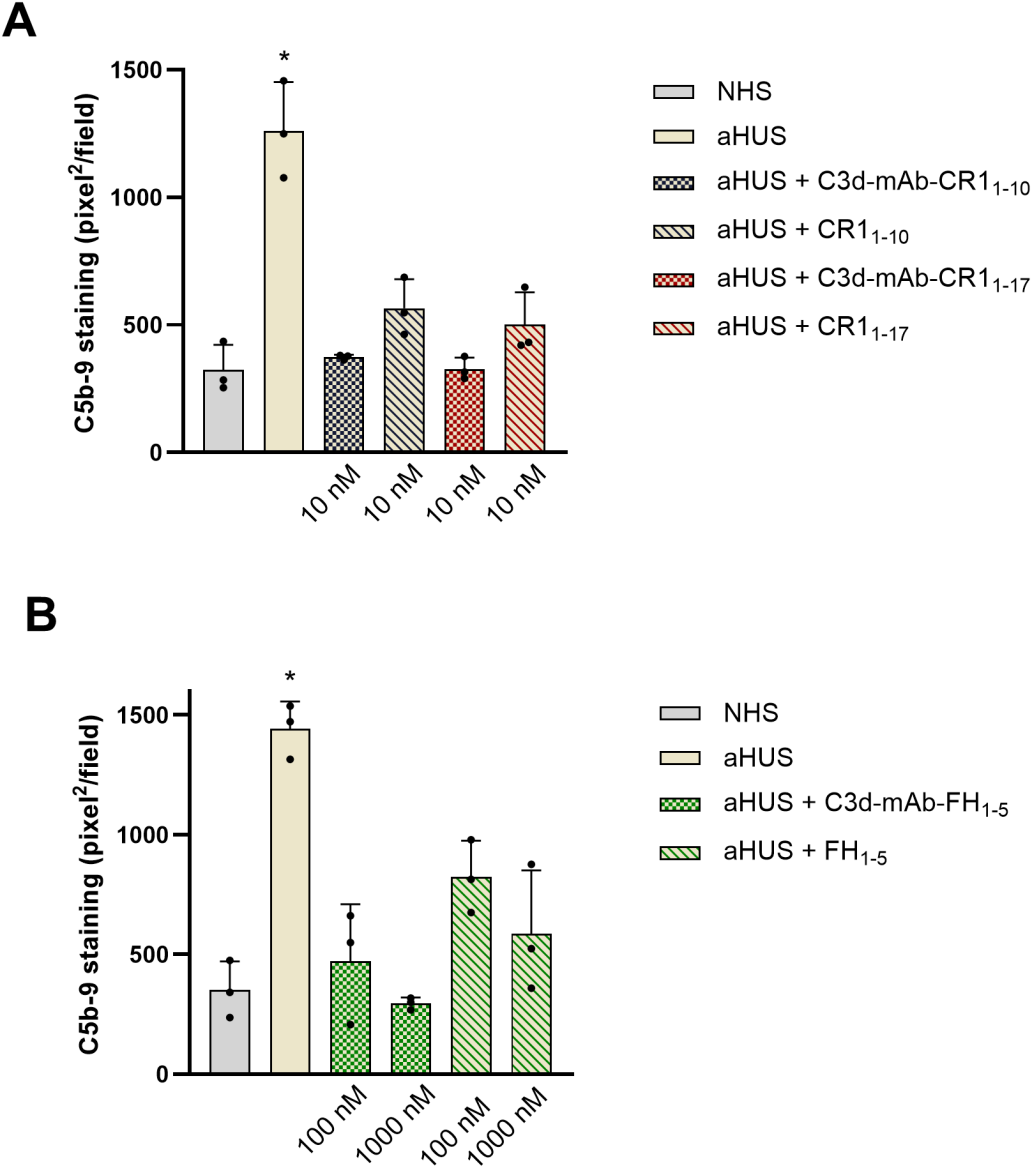
Effect of anti-C3d mAb conjugated with CR1_1–10_ or CR1_1-17_ (10 nM) and their corresponding non-targeted CR1_1-10_, CR1_1-17_ (10 nM) **(A)**; or anti-C3d mAb conjugated with FH_1-5_ (100 and 1000 nM) and its corresponding non-targeted FH_1-5_ (100 and 1000 nM) **(B)**, on C5b-9 formation on activated HMEC-1 cells exposed to serum from aHUS patients (one with RV in *CFH*, one with RV in *CFI*, and one with RV in *C3*). Data are expressed as area covered by C5b-9 staining (mean ± SD of three independent experiments). *p<0.05 vs all groups. NHS, normal human serum.

**Figure 6:**
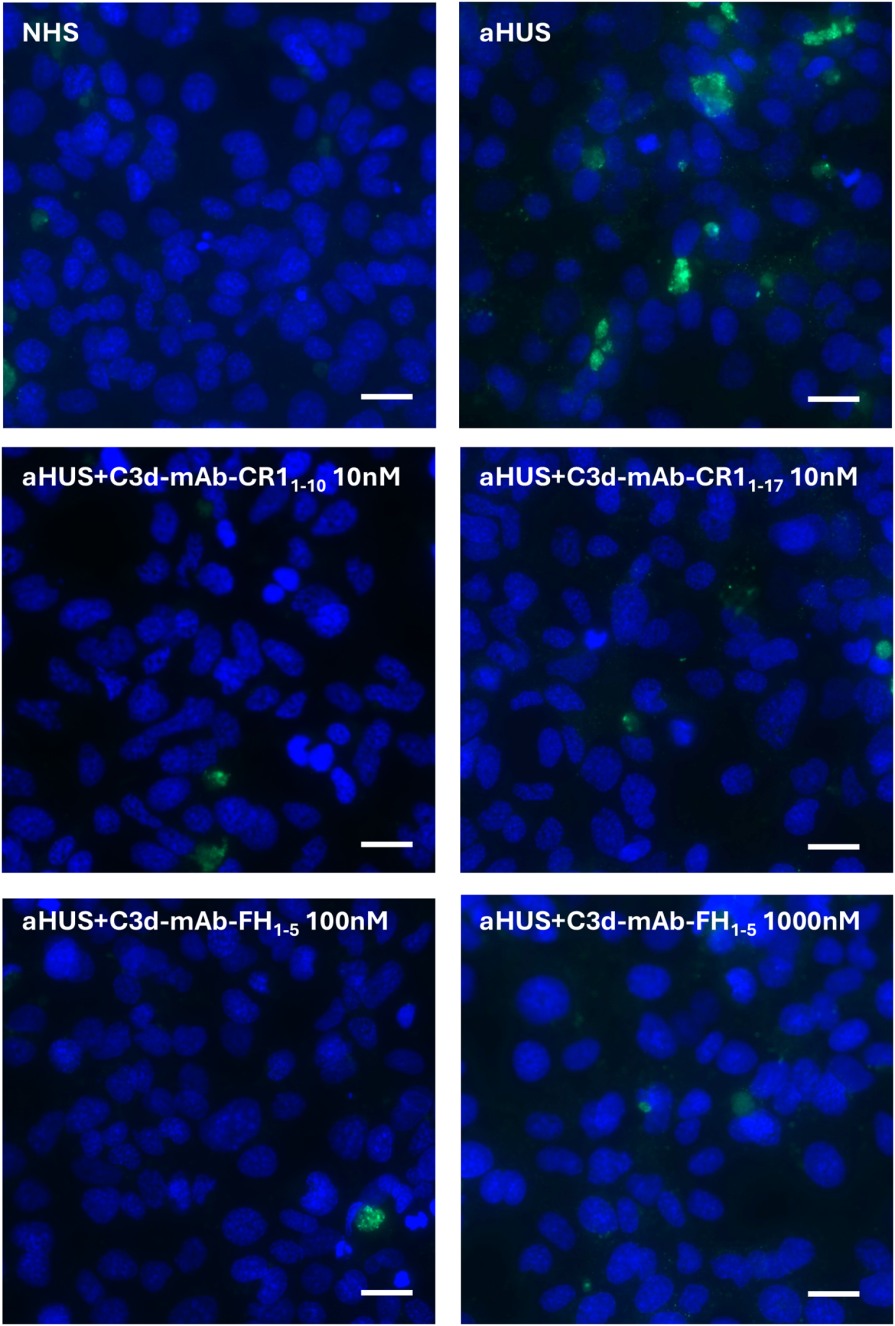
Representative images of C5b-9 staining (green) on activated HMEC-1 exposed to normal human serum (NHS) or serum from aHUS patients in the presence or absence of anti-C3d-CR1_1-10_ (10 nM), anti-C3d-CR1_1-17_ (10 nM), and anti-C3d-FH_1-5_ (100 and 1000 nM). Blue: DAPI. Scale bar: 20 µm.

Data showed a significant reduction of C5b-9 formation on cells exposed to sera added with either C3d-targeted CR1_1–10_ or CR1_1-17_ (**Figures 5A**, **6**), or C3d-targeted FH_1-5_ (**Figures 5B**, **6**), as well as with non-targeted CR1_1-10_, CR1_1-17_, or FH_1-5_ (**Figures 5A, B**). Notably, values of C5b-9 staining were comparable to those observed with control serum pool with C3d-targeted CR1_1–10_ and CR1_1–17_ at 10nM and with C3d-targeted FH_1–5_ at 1000 nM concentration (**Figures 5A, B**).

### Anti-C3d mAb conjugated with CR1_1–17_ or FH_1–5_ inhibit platelet adhesion and aggregation on HMEC-1 exposed to serum from aHUS patients

In aHUS complement activation on endothelial cell surface is associated with loss of anti-thrombotic properties (16). Thus, we then moved to evaluate the effect of C3d-targeted CR1_1–17_ and C3d-targeted FH_1–5_ on platelet adhesion and aggregation on HMEC-1 exposed to aHUS serum and then perfused with control whole blood. C3d-targeted CR1_1–17_ and FH_1–5_ were tested with sera from the same 3 patients selected for the above-described experiments of C5b-9 formation.

**Figure 7:**
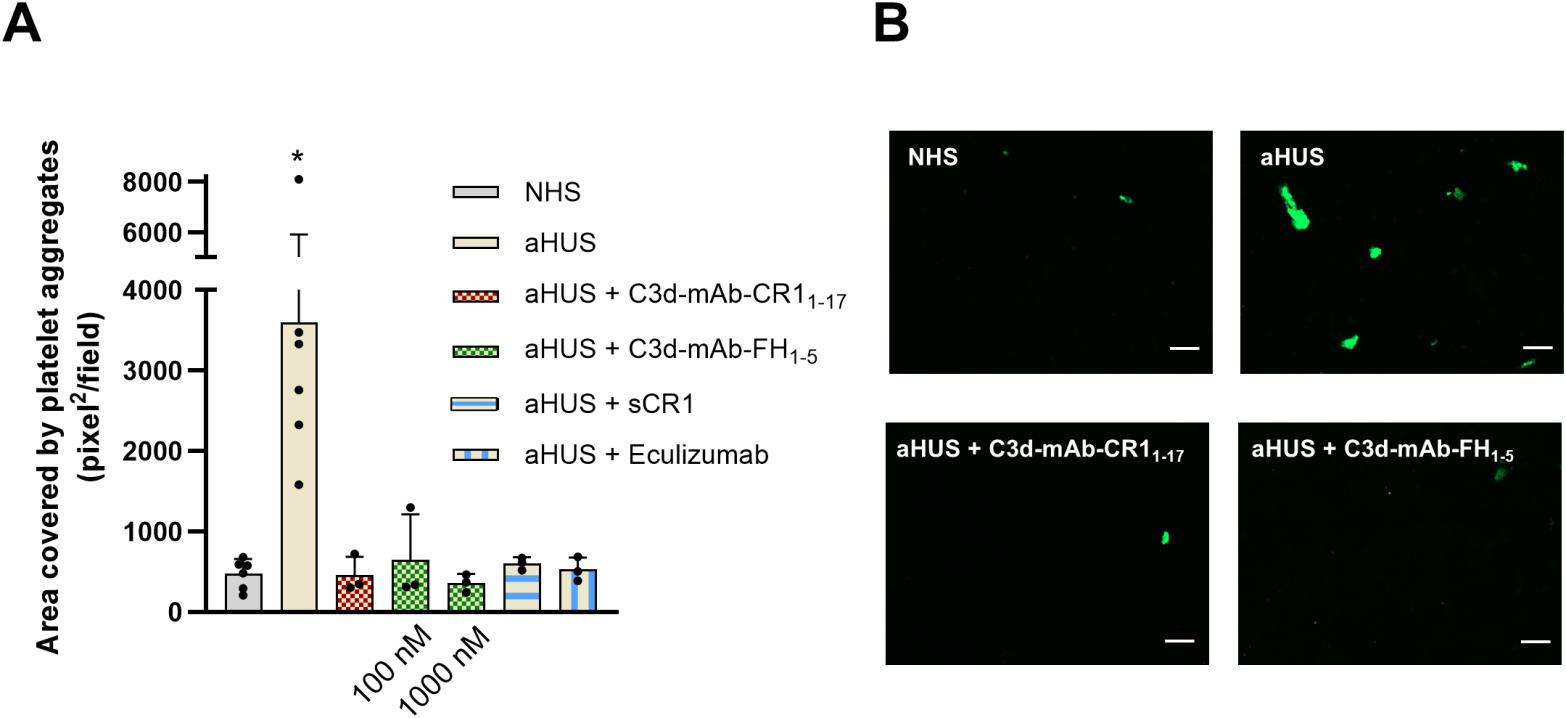
**(A)** Platelet adhesion and aggregation on activated HMEC-1 exposed to serum from aHUS patients (one with RV in *CFH*, one with RV in *CFI*, and one with RV in *C3*), in the presence or absence of anti-C3d mAb conjugated with CR1_1-17_ (10 nM), or anti-C3d mAb conjugated with FH_1-5_ (100 or 1000 nM), or sCR1 (150 µg/ml), or eculizumab (100 µg/ml), and then perfused with control whole blood. Data are expressed as area covered by platelet aggregates (mean ± SD). *p<0.05 vs all groups. **(B)** Representative images of experiments of platelet adhesion and aggregation (green staining) on HMEC-1 cells exposed to NHS, or aHUS serum with or without C3d-mAb-CR1_1-17_ (10 nM) or C3d-mAb-FH_1-5_ (1000 nM). NHS, normal human serum. Scale bar: 100 µm.

As shown in **Figure 7**, the area covered by platelet aggregates was about 10-fold wider on HMEC-1 pre-exposed to aHUS serum as compared with that observed on endothelium pre-exposed to control serum pool, confirming our previously published results (16). Addition of C3d-targeted CR1_1–17_ at 10 nM to aHUS serum fully prevented platelet adhesion and aggregation induced on HMEC-1 by the exposure to aHUS serum (**Figures 7A, B**). Similar effects were achieved by C3d-targeted FH_1-5_, at both 100 and 1000 nM (**Figures 7A, B**). The inhibitory effect was comparable to that achieved by the pan-complement inhibitor sCR1 as well as by the potent inhibitor of the complement terminal pathway, eculizumab.

## Discussion

Here we evaluated the effectiveness of anti-C3d antibodies -recognizing iC3b/C3dg/C3d- conjugated with fragments of endogenous complement inhibitors (CR1_1-10_, CR1_1–17_ or FH_1-5_) to correct complement dysregulation at the cell surface level. To assess this, we used ex-vivo assays that measure complement activation – C3 deposition and C5b-9 formation – induced by aHUS serum on microvascular endothelial cells. These assays have proven to be reliable in detecting complement dysregulation in aHUS patients, effectively replicating the endothelial-specific complement activation that characterizes the disease (14–19), and for evaluating the *in-vitro* efficacy of novel complement inhibitors (20).

Our findings confirm that exposure of microvascular endothelial cells to serum from aHUS patients results in abnormal deposition of active and degradation fragments of C3, and increased formation of C5b-9 (14–19). Importantly, we document that anti-C3d-conjugated CR1_1-10_, CR1_1-17_, and FH_1-5_, when added to aHUS serum, effectively prevented complement activation on endothelial cells, outperforming their soluble counterparts (CR1_1-10_, CR1_1-17_, and FH_1-5_). This highlights the potential of localizing complement endogenous negative regulators to C3d deposited on biologic surfaces as a promising strategy for cell/tissue-targeted complement inhibition, avoiding systemic blockade and thereby preserving the homeostatic functions of complement system (5). Furthermore, the observation that the inhibitory effects on complement activation on both C3/C3b and C5b-9 level, achieved with all the three C3d-targeted inhibitors, translated into complete prevention of platelet adhesion and aggregation on endothelial cells pre-exposed to aHUS serum, underscore their potential to counteract the direct consequence of complement activation on endothelium and highlight the potential clinical applicability of C3d-targeted CR1 and FH fragments for aHUS treatment.

Of relevance, we found that the effectiveness of tested compounds, varies according to the specific complement inhibitor conjugated to the anti-C3d antibody, and to the complement activation step being evaluated (C3 vs C5 convertase).

At low doses C3d-targeted CR1_1–17_ exerted more robust inhibition of aHUS serum-induced C3 deposits as compared to C3d-targeted CR1_1-10_. This finding may be interpreted considering that CR1 exerts its regulatory activities by binding C3b through two binding sites located within the regions comprising SCRs 8–11 and SCRs 15–18 (21), so that C3d-targeted CR1_1–10_ and CR1_1–17_ can bind one and two C3b molecules, respectively. In general, fragments of CR1 proved to be more effective than FH_1–5_ in inhibiting C3 deposition, while both strategies were equally effective in inhibiting C5b-9 formation. The AP C5 convertase is formed by the addition of extra C3b molecules to the C3 convertase, and its activity relies heavily on the density of C3b on cell surfaces (22). Thus, even partial inhibition of C3b deposition may be enough to effectively block C5 convertase. Finding that, even an uncomplete inhibition of C3 convertase is sufficient to penetrate to C5 convertase level and translate to anti-thrombotic activity, is consistent with the above hypothesis.

Another finding of this study is that the complement inhibitory effectiveness of C3d-targeted complement regulators is influenced by the specific genetic defect carried by the patient. All three C3d-targeted complement inhibitors required higher concentration to effectively correct complement dysregulation in the presence of RVs affecting the SCRs 19–20 of *CFH*. These SCRs contain a C3b-binding site and a polyanion-binding site that are crucial for CFH binding and complement regulatory activity on cell surfaces (23). As a consequence, mutations in these regions have a profound impact on CFH regulatory effect (23).

On the other hand, C3d-targeted FH_1–5_ corrected C3 convertase dysregulation more effectively than soluble FH_1–5_ in samples from two patients: one carrying the p.R78G RV in *CFH* and the other carrying the p.S1063R RV in *C3*. These findings may be interpreted as to indicate that increasing the local concentration of functional FH_1–5_ by C3d-anchoring helps compensate for the reduced C3b-CFH binding affinity associated with these RVs (24, 25).

Preclinical studies have recently tested the potential therapeutic effect of C3d-targeted CR1_1–10_ and FH_1–5_ in C3 glomerulopathies (C3G) by studying their tissue localization and complement inhibition effectiveness in CfH knockout mice (CfH^-/-^) (9, 10). These mice exhibit uncontrolled systemic complement activation, which results in a 10- to 20-fold reduction in circulating intact C3 protein levels, and increased C3 deposition in the liver and kidneys. This leads to complement-mediated kidney injury, closely mimicking the clinical features of C3G (10, 26). Both C3d-targeted CR1_1–10_ and C3d-targeted FH_1-5_, administered to CfH^-/-^ mice at low doses, effectively localized to injured tissues and reduced C3 fragments deposition for durations exceeding 10 days (9, 10), with circulating levels of the fusion proteins remaining below 3 µg/ml. Notably at these low doses, unlike soluble FH_1-5_, treatment of CfH^-/-^ mice with C3d-targeted FH_1–5_ resulted in marked and sustainable complement inhibition in both the liver and kidneys, while avoiding circulating complement blockade as documented by only a negligible increase in intact C3 plasma levels after drug administration (10). In addition, C3d-targeted FH_1–5_ treatment achieved similar effects when administered either intravenously or subcutaneously (10), with the subcutaneous route offering the advantage of an easier administration in potential human applications.

Beyond aHUS and C3G, persistent, uncontrolled complement activation plays a major role in several other diseases, including paroxysmal nocturnal hemoglobinuria (PNH) (27). Similar to aHUS patients, those with PNH are treated with anti-C5 therapies. However, some patients on these therapies experience frequent C3-mediated extravascular hemolysis flares (28). Current guidelines recommend switching these patients to therapies that inhibit complement cascade upstream, such as the recently approved C3 inhibitor, pegcetacoplan (29). Nevertheless, like anti-C5 blockade, C3-blocking therapies carry a significant risk of serious infections, which supports the potential of local cell membrane C3d-targeted complement inhibitors as a safer alternative therapeutic approach for PNH patients who do not respond adequately to anti-C5 therapies. The possibility that the fusion proteins described here might recognize certain pathogens opsonized with iC3b, C3dg, or C3d cannot be excluded based on the current data. However, previous studies have shown that both C3d-targeted CR1_1–10_ and C3d-targeted FH_1–5_ influenced complement-mediated hemolysis of rabbit erythrocytes—used as a model for complement-activating surfaces—only at concentrations far exceeding those effective in rodent models of complement-mediated diseases (9, 10). These findings support the potential of the two fusion proteins to control tissue-localized complement activation effectively, without impairing systemic antibacterial responses.

In summary, despite the limitations posed by the small sample size, our findings indicate that targeting complement inhibitors to C3d effectively corrects complement dysregulation on endothelial cells in aHUS patients. While the efficacy of C3 convertase inhibition varied depending on the complement inhibitor used (CR1_1-17_ > CR1_1-10_ > FH_1-5_) and the specific genetic abnormalities of the patients, all the inhibitors successfully blocked C5 convertase activity and eliminated the pro-thrombogenic effects of aHUS patients’ serum. These results highlight the potential of tissue-targeted complement inhibition, paving the way to new therapeutic approaches that are effective at low doses without affecting systemic complement activity. This approach may benefit aHUS as well as other diseases characterized by cell/tissue-restricted complement activation.

## Data Availability

The raw data supporting the conclusions of this article will be made available by the authors, without undue reservation.
