# Supplementary material for: C3d-targeted complement inhibitors to correct complement dysregulation in aHUS patients
Source: Front Immunol. 2025 Jun 20;16:1620996. doi: 10.3389/fimmu.2025.1620996 (PMC12226290; doi:10.3389/fimmu.2025.1620996)
Supplement: Supplementary file 1 [file DataSheet1.pdf]

C3d-targeted complement inhibitors to correct complement dysregulation in aHUS patients

Valeria Guaschino[1]*, Donata Santarsiero[1]*, Sara Gastoldi[1], Joshua M. Thurman[2], V. Michael Holers[2], Shelia M. Violette[3], Fei Liu[3], Kelly C. Fahnoe[3], Chiara Guarinoni[1], Ariela Benigni[1], Giuseppe Remuzzi[1], Marina Noris[1], Sistiana Aiello[1]

Supplementary material:

Supplementary Figure 1

Supplementary Figure 2

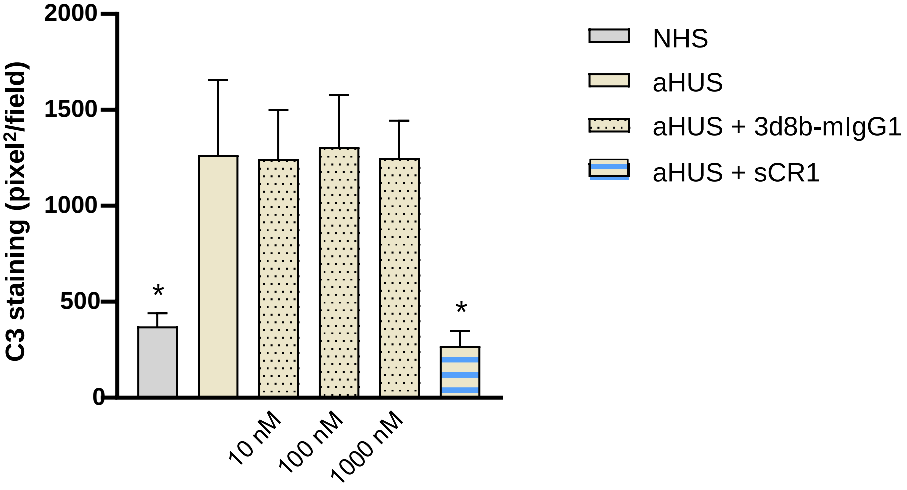

C3 deposition on ADP-activated HMEC-1 exposed to aHUS serum added with anti-C3d mAb (3d8b-mIgG1) (10, 100 and 1000 nM) or added with the pan-complement inhibitor sCR1 (150 µg/ml). Sera from nine aHUS patients (three with CFH rare variants, three with CFI rare variants, and three with C3 rare variants) have been evaluated. Data are expressed as area covered by C3 staining (mean ± SD of nine indipendent experiments). * $p<0.05$ vs aHUS, aHUS + 3d8-mIgG1 (10, 100, 1000 nM). NHS: normal human serum.

Supplementary Figure 1

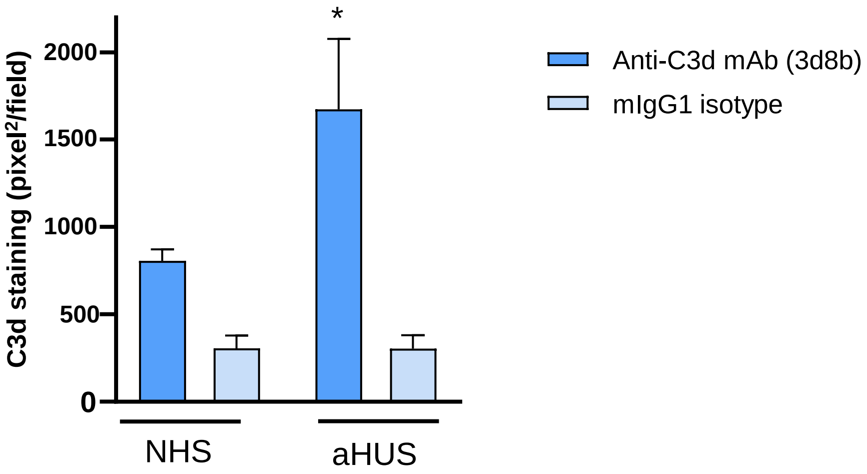

C3d deposition induced on activated HMEC-1 by serum from aHUS patients carrying rare gene variants in CFH (n=1), or CFI (n=1) or C3 (n=1). Data are expressed as area covered by C3d staining measured by anti-C3d 3d8b-mAb (mean ± SD, three independent experiments). *****$p<0.05$ vs NHS.

Supplementary Figure 2
